# Synthesis and Surface Strengthening Modification of Silica Aerogel from Fly Ash

**DOI:** 10.3390/ma17071614

**Published:** 2024-04-01

**Authors:** Lei Zhang, Qi Wang, Haocheng Zhao, Ruikang Song, Ya Chen, Chunjiang Liu, Zhikun Han

**Affiliations:** 1 School of Geology and Environment, Xi’an University of Science and Technology, Xi’an 710054, China; 18391871932@163.com (Q.W.); haocheng202302@163.com (H.Z.); srk781388595@163.com (R.S.); c1431332271@163.com (Y.C.); leocj2000@163.com (C.L.); han619332950@163.com (Z.H.); 2 Key Laboratory of Coal Resources Exploration and Comprehensive Utilization, Ministry of Natural Resources, Xi’an 710021, China

**Keywords:** fly ash, silica aerogel, acid dissolution–alkali leaching, ambient pressure drying, reinforcing modification

## Abstract

This study focuses on using activated fly ash to preparate silica aerogel by the acid solution–alkali leaching method and ambient pressure drying. Additionally, to improve the performance of silica aerogel, C_6_H_16_O_3_Si (KH-570) and CH_3_Si(CH_3_O)_3_ (MTMS) modifiers were used. Finally, this paper investigated the factors affecting the desilication rate of fly ash and analyzed the structure and performance of silica aerogel. The experimental results show that: (1) The factors affecting the desilication rate are ranked as follows: hydrochloric acid concentration > solid–liquid ratio > reaction temperature > reaction time. (2) KH-570 showed the best performance, and when the volume ratio of the silica solution to it was 10:1, the density of silica aerogel reached a minimum of 183 mg/cm^3^. (3) The optimal process conditions are a hydrochloric acid concentration of 20 wt%, a solid–liquid ratio of 1:4, a reaction time of two hours, and a reaction temperature of 100 °C. (4) The optimal performance parameters of silica aerogel were the thermal conductivity, specific surface area, pore volume, average pore size, and contact angle values, with 0.0421 W·(m·K)^−1^, 487.9 m^2^·g^−1^, 1.107 cm^3^·g^−1^, 9.075 nm, and 123°, respectively. This study not only achieves the high-value utilization of fly ash, but also facilitates the effective recovery and utilization of industrial waste.

## 1. Introduction

Fly ash is an industrial waste that is difficult to effectively utilize and is often stored in piles. This not only takes up a large amount of land, but also poses harm to humans and the environment when harmful substances migrate to the soil, atmosphere, and water [1,2]. But, the fly ash can still be utilized in various ways. Presently, its primary bulk application lies in the manufacturing of construction materials [3,4,5,6,7]. Nevertheless, fly ash is also rich in chemical elements such as Si, Al, Fe, and C, which constitutes a significant rationale for exploring high-value utilization pathways for fly ash [8,9,10,11,12]. Extracting and utilizing these chemical elements from fly ash can truly achieve the high-value utilization of fly ash [8,13]. Therefore, the comprehensive utilization of fly ash has important ecological and socio-economic benefits. 

Aerogel is a porous solid material composed of nanoparticles with a three-dimensional network structure [14]. It commonly includes materials such as silica aerogel and Al_2_O_3_ aerogel [15,16,17,18]. Silica aerogel, in particular, has gained attention due to its high porosity, high specific surface area, low density, and low thermal conductivity, and these characteristics make it suitable for applications in industrial insulation materials, electrical fields, acoustic fields, and environmental fields [19,20,21,22,23,24].

Currently, the preparation methods for silica aerogel typically involve the sol–gel method, aging method, solvent replacement method, and drying process [25,26,27,28]. However, the use of expensive organic alkoxides as silicon sources and the high cost, danger, and complexity associated with the supercritical drying method limit its large-scale industrial application [17,29,30,31,32]. Therefore, it is significant to explore low-cost, efficient, and environmentally friendly silicon sources and safe, cost-effective drying methods for the preparation of silica aerogel.

Considering the high-silica (SiO_2_) content in fly ash, it can be used as a raw material for the preparation of silica aerogel. The utilization of fly ash through methods such as acid dissolution, alkali leaching, sol–gel processing, surface modification, and atmospheric drying can effectively reduce the production cost of preparing silica aerogels from fly ash, while also offering new avenues for the utilization of fly ash [15,33,34,35,36]. Consequently, the utilization of fly ash and the preparation of silica aerogel using fly ash as a raw material have the potential to reduce costs, increase value-added utilization, and have significant ecological and socio-economic benefits. However, further research is required to optimize the preparation methods and achieve aerogel with desired properties.

In the first step of this study, the sodium silicate solution was prepared from activated fly ash by the acid dissolution and alkali leaching method. During this process, the single-factor effects of different experimental conditions (the HCl mass fraction, reaction temperature, reaction time, and solid–liquid ratio) on the desilication rate of fly ash were investigated. Subsequently, the interaction effects of four experimental conditions on the desilication rate of fly ash were studied through an orthogonal test, and the optimal experimental conditions for preparing a sodium silicate solution from fly ash were determined. In the second step, silica aerogels were prepared using the sodium silicate solution prepared under the optimal experimental conditions through processes such as the sol–gel process and ambient pressure drying. Additionally, to enhance the performance of silica aerogels, a surface enhancement modification process was introduced during the preparation, and the modification mechanisms of different modifiers (KH-570 and MTMS) were investigated. Eventually, surface-enhanced modifiers with excellent performance were selected based on the experimental results.

**Figure 1:**
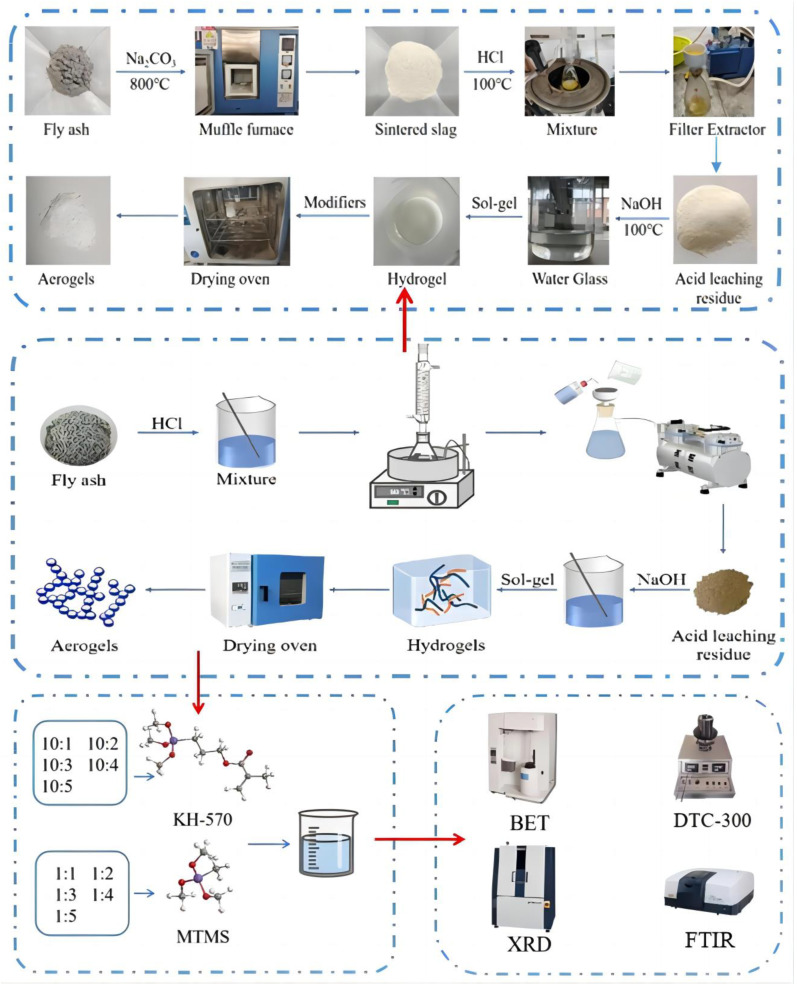
Experimental schematic diagram of preparation and modification of silica aerogel.

Finally, to analyze the microstructure, functional groups, crystalline phases, thermal conductivity, hydrophobicity, and specific surface area of the silica aerogels, the characterizations of the composition and properties of the silica aerogels were conducted using X-ray Diffraction (XRD), a Fourier Transform Infrared Spectrometer (FT-IR), Brunauer–Emmett–Teller (BET), contact angle measurement, and a thermal conductivity meter. Meanwhile, the changes in the composition and properties of the silica aerogels during the preparation process were studied to explain the reaction mechanism of preparing silica aerogels by ambient pressure drying. The experimental principle flow chart is shown in Figure 1.

## 2. Experiment

### 2.1. Materials

The fly ash utilized in the experiment is derived from a coal-fired furnace. Table 1 displays the chemical composition of the fly ash employed in the study. As observed, the SiO_2_ and Al_2_O_3_ content in this particular fly ash surpasses 80%, with SiO_2_ constituting over 50% of the composition.

### 2.2. Characterization Methods

The main experimental equipment as shown Supplementary Table S2.

#### 2.2.1. XRD

XRD analysis was carried out using an XD-3X (Beijing Puxi General Instrument Co., Ltd., Beijing, China). Prior to testing, SiO_2_ aerogel is ground into powder. The initial angle is 5°, the final angle is 90°, the step width is 0.02, and the wavelength is 1.54, utilizing Cu target Kα radiation, with an operating voltage of 36 kV, and an operating current of 20 mA.

#### 2.2.2. BET

BET analysis was carried out using an ASAP2020 (Mike, Detroit, MI USA). Prior to measurement, samples are degassed under vacuum conditions at 200 °C for 6 h. Subsequently, high-purity nitrogen is used as the adsorbate, and adsorption–desorption measurements are conducted at 77 K.

#### 2.2.3. FTIR

FTIR analysis was carried out using a VERTEX70 (Bruker, Karlsruhe, Germany) after grinding SiO_2_ aerogel. Samples are prepared by mixing with KBr and pressing into pellets for testing and analysis. The measurement range is 400~4000 cm^−1^, with 28 scans, and a resolution of 0.4 cm^−1^.

#### 2.2.4. Contact Angle

Contact angle analysis was carried out using a CA100B (Shanghai Yingnuo Precision Instrument Co., Ltd., Shanghai, China), using the sessile drop method at room temperature.

#### 2.2.5. Thermal Conductivity

Thermal conductivity analysis was carried out using a TC-3000E (Xi’an Xiaxi Electronic Technology Co., Ltd., Xi’an, China), pressing the SiO_2_ aerogel into blocks (Thickness ≥ 0.1 mm, Length ≥ 25 mm) at room temperature.

### 2.3. Preparation of Silica Aerogel Modified Silica Aerogel

#### 2.3.1. Extraction of SiO_2_ through Acid Dissolution and Alkali Leaching from Fly Ash

In this processing stage, Si-Al bonds in fly ash are initially disrupted using an acid dissolution method, followed by alkali leaching to extract SiO_2_ and prepare a sodium silicate solution. The experimental reagents as shown Supplementary Table S1.

1. Acid Dissolution: Pre-treated fly ash is mixed with hydrochloric acid solutions of varying mass fractions (10 wt%, 15 wt%, 20 wt%, 25 wt%, and 30 wt%) in specific ratios (1:3, 1:4, 1:5, 1:6, and 1:7). The mixture is stirred in a magnetic stirrer at a designated temperature (80 °C, 90 °C, 100 °C, 110 °C, and 120 °C) for a specified duration (1 h, 2 h, 3 h, 4 h, and 5 h). After completion, the reaction mixture is filtered and repeatedly washed with distilled water until neutral. The residue is then dried at 105 °C for 3–5 h in a vacuum drying oven.
2. Alkali Leaching: The filtered residue is mixed with sodium hydroxide solutions of various mass fractions (10 wt%, 15 wt%, and 20 wt%) in a 1:5 ratio. The mixture is stirred in a magnetic stirrer at a certain temperature (80 °C, 90 °C, and 100 °C) for a specific duration (1 h, 1.5 h, and 2 h). After completion, the mixture is filtered and distilled water is added for washing until neutral. The filtrate is collected and stored in a beaker, representing the NaSiO_3_ solution.

#### 2.3.2. Preparation of Strengthened and Modified Silica Aerogel

In this processing stage, modified silica aerogels are prepared through the processes of wet gel formation, surface enhancement modification, aging, and ambient pressure drying.

1. Preparation of wet gel and surface enhancement modification: The sodium silicate solution obtained from fly ash is poured into an ion exchange column containing strongly acidic cation exchange resin, resulting in a silicon acid solution with a pH of 2–3. Modified liquid is prepared by mixing MTMS and KH-570 with anhydrous ethanol in a 1:4 ratio. The silicon acid solution is mixed with the modified liquid in a certain proportion and stirred for 1 h. After stirring, the pH of the mixed solution is adjusted to 5–6 using 1 mol/L ammonia solution. The sealed gel is left to age, observed by tilting the beaker at a 45° angle to check for gel formation.
2. Aging: deionized water is added to the beaker containing the wet gel, sealed, and placed in a 50 °C water bath for aging for 48 h.
3. Solvent replacement: the aged wet gel is soaked in anhydrous ethanol and placed in a 50 °C water bath for solvent replacement for 24 h.
4. Post-treatment modification: the wet gel, after solvent replacement, is soaked in a mixture of n-hexane, HMDSO, and ethanol (with HMDSO to wet gel volume ratio of 1:1) at 50 °C for surface modification for 24 h.
5. Solvent replacement: the modified gel is soaked in n-hexane and placed in a 50 °C water bath for solvent replacement for 12 h.
6. Ambient pressure drying: the gel, after solvent replacement, is placed in a vacuum drying oven and dried at 60 °C, 80 °C, 120 °C, and 180 °C for 2 h each, resulting in strengthened and modified SiO_2_ aerogels.

### 2.4. The Influence of Acid Leaching Conditions on the Desilication Rate of Fly Ash 

Mix the fly ash thoroughly with hydrochloric acid and allow it to react. Once the reaction is complete, filter the mixture while it is still hot and then proceed to wash the filter residue. Subsequently, add 20% sodium hydroxide solution to the dried acid leaching residue at a ratio of 1:5. Place the mixture in a magnetic stirrer and allow it to react for 2 h at a temperature of 100 °C. After the reaction, filter the mixture using hot water and wash it until it reaches a neutral state. The resulting filtrate is a sodium metasilicate solution. Finally, select appropriate reaction conditions for the orthogonal test using an L9 (3^4^) orthogonal design table. The experimental conditions include the mass fraction of hydrochloric acid (15%, 20%, and 25%), solid–liquid ratio (1:3, 1:4, and 1:5), reaction temperature (90 °C, 100 °C, and 110 °C), and reaction time (2 h, 3 h, and 4 h). Through these experiments, we investigated the impact of these four factors on the desilication rate.

## 3. Results and Discussion

### 3.1. Effect of Acid Leaching Conditions on the Desilication Rate of Fly Ash

After acid leaching, the fly ash undergoes a certain level of activation, leading to the formation of a significant amount of amorphous SiO_2_ as a result of mullite structure disruption. The reaction equation is as follows:

(1) $${\mathrm{NaAlSiO}}_{4}+4\mathrm{HCl}\to \mathrm{NaCl}+{\mathrm{AlCl}}_{3}+{\mathrm{SiO}}_{2}+2H_{2}O$$

#### 3.1.1. Effect of Hydrochloric Acid Concentration on the Desilication Rate of Fly Ash

**Figure 2:**
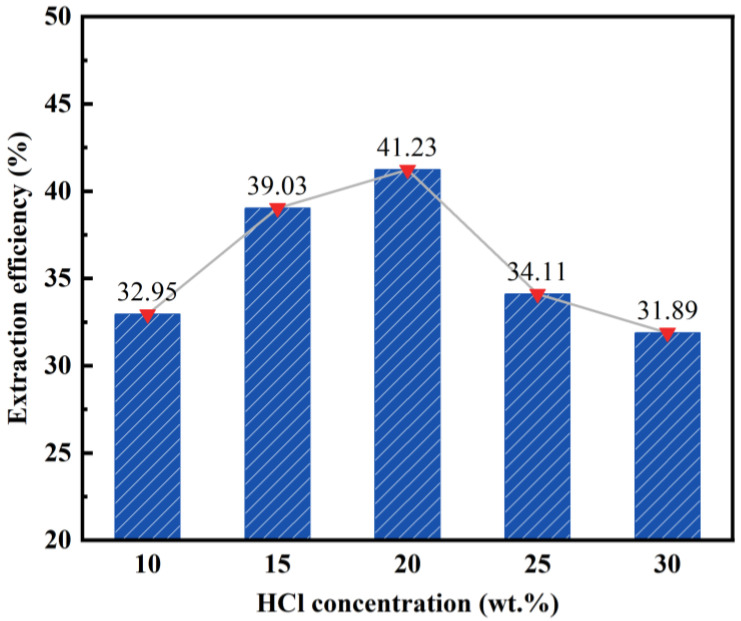
Effect of HCl concentration on the desilication rate of coal fly ash.

By maintaining a constant solid–liquid ratio (1:4), reaction temperature (100 °C), and reaction time (2 h), we can investigate the impact of varying hydrochloric acid concentrations on the desilication rate of fly ash. Figure 2 illustrates the obtained results.

According to the findings presented in Figure 2, the desilication rate of fly ash exhibits an initial increase followed by a decrease with the rise in the hydrochloric acid concentration. At a concentration of 10 wt%, the desilication rate reaches its lowest point at 31.89%. This can be attributed to the insufficient reaction between hydrochloric acid and SiO_2_ in fly ash caused by the low acid concentration. Conversely, at a concentration of 20 wt%, the desilication rate peaks at 41.23%. However, as the concentration of the hydrochloric acid exceeds 20–30 wt%, the desilication rate gradually declines. This decline may be attributed to side reactions occurring between the hydrochloric acid and other substances present in fly ash due to the high acid concentration. These side reactions increase the overall amount of reactants and consequently reduce the desilication rate.

#### 3.1.2. Effect of Solid–Liquid Ratio on the Desilication Rate of Fly Ash

**Figure 3:**
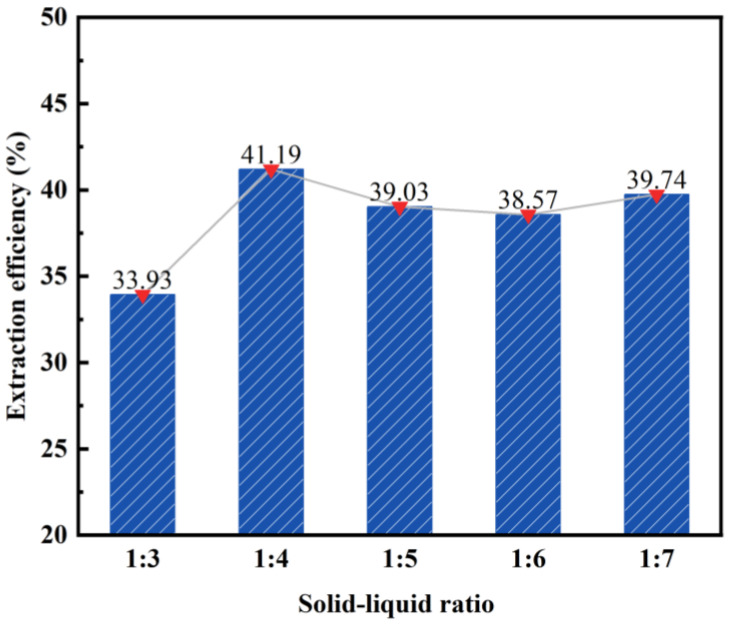
Effect of solid–liquid ratio on the desilication rate of coal fly ash.

By maintaining a constant hydrochloric acid concentration (20 wt%), reaction temperature (100 °C), and reaction time (2 h), we can explore the impact of varying solid–liquid ratios on the desilication rate of fly ash. The obtained results are illustrated in Figure 3.

According to the data presented in Figure 3, the desilication rate of fly ash initially increases and then stabilizes as the solid–liquid ratio decreases. At a solid–liquid ratio of 1:3, the desilication rate reaches its lowest point at 33.93%. This can be attributed to the insufficient addition of hydrochloric acid, resulting in an inadequate reaction between the fly ash and hydrochloric acid. Conversely, at a solid–liquid ratio of 1:4, the highest desilication rate is observed at 41.19%. At this ratio, the reaction between the fly ash and hydrochloric acid is deemed complete. With further increases in the solid–liquid ratio, specifically reaching 1:7, there is no significant change in the desilication rate of fly ash. Consequently, a solid–liquid ratio of 1:4 was chosen as the subsequent reaction condition.

#### 3.1.3. Effect of Reaction Temperature on the Desilication Rate of Fly Ash

**Figure 4:**
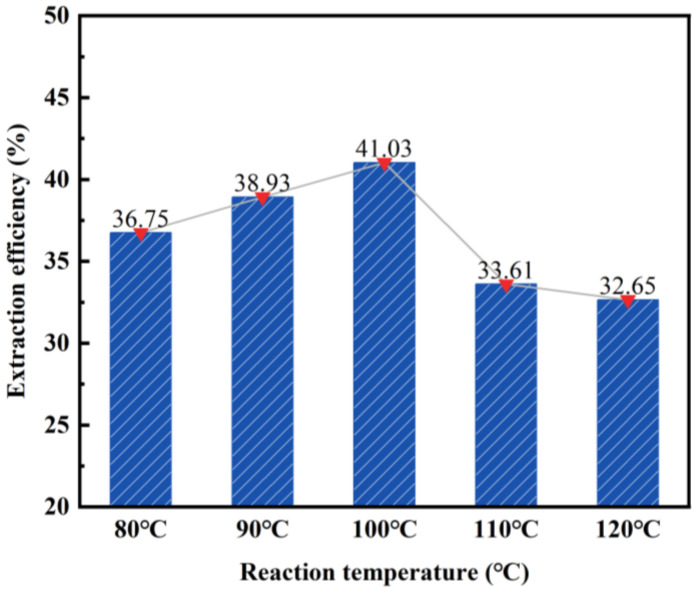
Effect of reaction temperature on the desilication rate of fly ash.

By maintaining a constant hydrochloric acid concentration (20 wt%), solid–liquid ratio (1:4), and reaction time (2 h), we can investigate the impact of varying reaction temperatures on the desilication rate of fly ash. The obtained results are illustrated in Figure 4.

According to the findings presented in Figure 4, the desilication rate of fly ash initially increases and then decreases as the reaction temperature increases. A turning point is observed at 100 °C, with the highest desilication rate reaching 41.03%. However, the desilication rate is found to be the lowest at 120 °C, measuring 32.65%. The primary reason for this is that the boiling point of 20 wt% hydrochloric acid is 110 °C. When the temperature exceeds 110 °C, the solution reaches its boiling point and undergoes rapid evaporation, thereby affecting the acid-leaching effect of fly ash. Consequently, a reaction temperature of 100 °C was chosen as the subsequent reaction condition.

#### 3.1.4. Effect of Reaction Time on the Desilication Rate of Fly Ash

**Figure 5:**
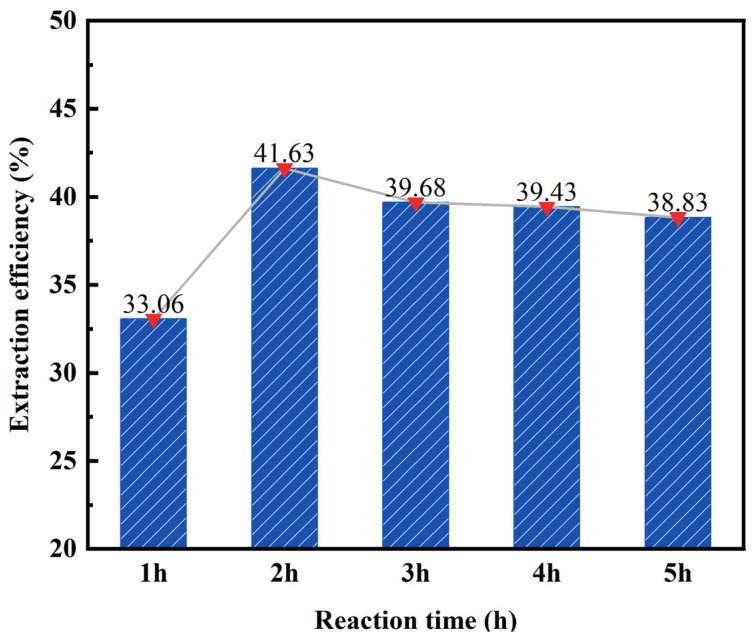
Effect of reaction time on the desilication rate of fly ash.

By maintaining a constant hydrochloric acid concentration (20 wt%), solid–liquid ratio (1:4), and reaction temperature (100 °C), we can explore the impact of varying reaction times on the desilication rate of fly ash. The obtained results are illustrated in Figure 5.

According to the data presented in Figure 5, the desilication rate of fly ash initially increases and then stabilizes as the reaction time increases. At a reaction time of 1 h, the desilication rate reaches its lowest point at 33.06%. This can be attributed to the short reaction time, which hinders the complete reaction between the fly ash and hydrochloric acid. Conversely, at a reaction time of 2 h, the highest desilication rate is observed at 41.19%, indicating that the acid leaching reaction is completed at this point. When the reaction time is extended to 5 h, there is no significant change in the desilication rate. Consequently, a reaction time of 2 h was chosen as the subsequent reaction condition.

#### 3.1.5. Interactive Effects of Different Reaction Conditions on the Desilication Rate of Fly Ash

To investigate the combined effect of reaction conditions on the desilication rate of fly ash while keeping the alkali-leaching reaction conditions constant, four factors were chosen for the test, as shown in Table 2. Each factor was tested at three different levels. The details of the orthogonal test results and the corresponding analysis can be found in Table 3 and Table 4. The orthogonal test results analysis table as shown Supplementary Table S3.

According to the data provided in Table 4, the hydrochloric acid concentration exhibits the largest range of 5.07, indicating that it has the most significant impact on the desilication rate of fly ash. In contrast, the reaction time shows the smallest range of 1.62, suggesting that its effect on the desilication rate is relatively minor. The solid–liquid ratio demonstrates a range of 5.03, indicating that both the hydrochloric acid concentration and solid–liquid ratio have a substantial influence on the desilication rate. Thus, the order of importance for the four influencing factors is as follows: the hydrochloric acid concentration > solid–liquid ratio > reaction temperature > reaction time. Based on the k-value analysis, the optimal conditions for the desilication of fly ash are as follows: a hydrochloric acid concentration of 20 wt%, a solid–liquid ratio of 1:4, a reaction temperature of 100 °C, and a reaction time of 2 h.

#### 3.1.6. Optimization of Acid Leaching Conditions

The desilication rate of fly ash is limited to approximately 42% under the optimal acid solution alkali leaching condition. Consequently, to enhance the activity of fly ash, a method involving the activation of fly ash through baking with sodium carbonate is employed to generate a sodium metasilicate solution, which ultimately leads to a higher desilication rate. The reaction equation is as follows:

(2) $$3{\mathrm{Al}}_{2}O_{3}\cdot 2{\mathrm{SiO}}_{2}+4{\mathrm{SiO}}_{2}+3{\mathrm{Na}}_{2}{\mathrm{CO}}_{3}\to 6{\mathrm{NaAlSiO}}_{4}+3{\mathrm{CO}}_{2}$$

(3) $${\mathrm{NaAlSiO}}_{4}+4\mathrm{HCl}\to \mathrm{NaCl}+{\mathrm{AlCl}}_{3}+{\mathrm{SiO}}_{2}+2H_{2}O$$

(4) $${\mathrm{SiO}}_{2}+2\mathrm{NaOH}={\mathrm{Na}}_{2}{\mathrm{SiO}}_{3}+H_{2}O$$

**Figure 6:**
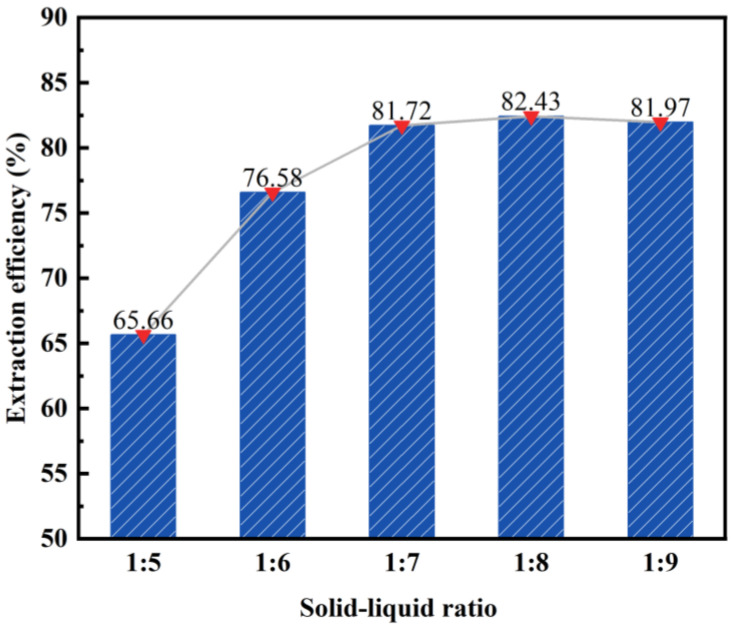
Effect of solid–liquid ratio on the desilication rate of activated fly ash.

During the acid leaching experiment, the reaction unexpectedly ceased as a result of fly ash agglomeration. Further investigation, as indicated by a previous study, revealed that the incomplete reaction was due to an excessively low solid–liquid ratio of fly ash to hydrochloric acid [33]. To address this issue, the experimental conditions were maintained at a constant (Ceteris paribus), and the influence of different solid–liquid ratios on the desilication rate of fly ash was investigated. The results of this study can be observed in Figure 6.

As depicted in Figure 6, the desilication rate of fly ash exhibits an initial increase followed by stabilization as the solid–liquid ratio decreases. Notably, when the solid–liquid ratio is 1:5, the desilication rate reaches its lowest point at 65.66%. This is primarily attributed to the inadequacy of hydrochloric acid, which hinders the complete reaction between fly ash and hydrochloric acid. Conversely, when the solid–liquid ratio is 1:7, the highest desilication rate of 81.72% is achieved. At this point, the reaction between fly ash and hydrochloric acid is completed. The further reduction of the solid–liquid ratio to 1:9 does not yield any significant change in the desilication rate. Consequently, after careful consideration, a solid–liquid ratio of 1:7 was selected as the subsequent reaction condition.

### 3.2. Surface Strengthening Modification of Silica Aerogel

#### Effect of Modifier on the Density of Silica Aerogel

**Figure 7:**
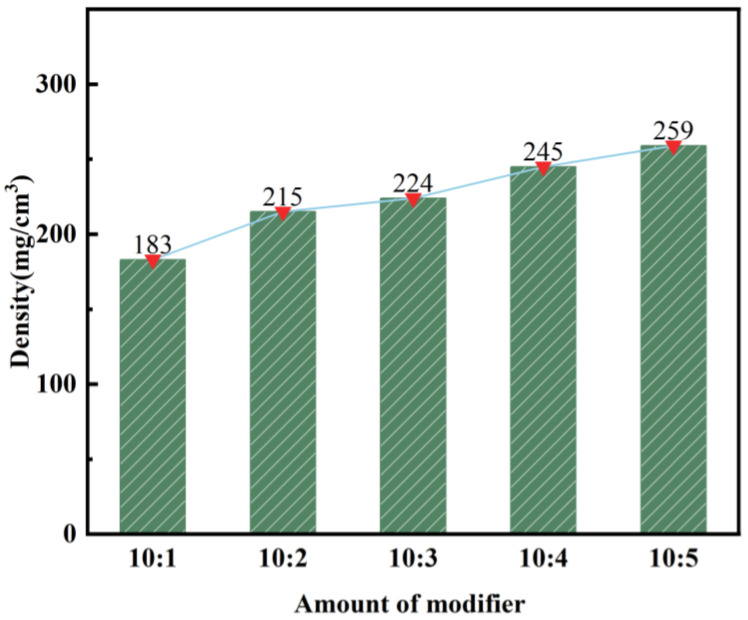
Effect of KH-570 dosage on the density of silica aerogel.

**Figure 8:**
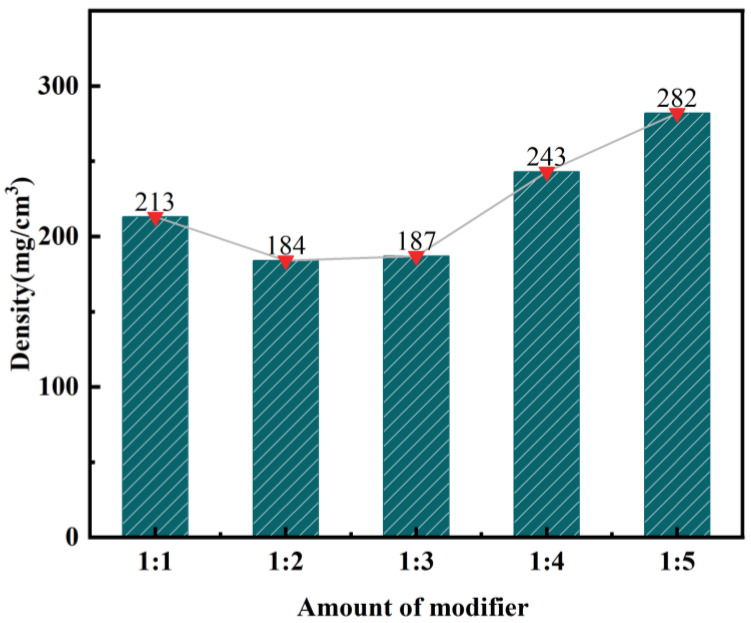
Effect of MTMS dosage on the density of silica aerogel.

This section focuses on analyzing the process of producing modified silica aerogel from calcined, activated fly ash. The objective is to investigate the impact of different modifiers on the density of the aerogel. The ratio of the modified solution can be found in Table 5, while the corresponding experimental results are illustrated in Figure 7 and Figure 8.

As depicted in Figure 7, the density of silica aerogel shows an increasing trend with the addition of KH-570. Specifically, at a volume ratio of 10:1 for the silicic acid solution to KH-570, the minimum density is recorded at 183 mg/cm^3^. Conversely, at a volume ratio of 10:5, the maximum density reaches 259 mg/cm^3^. This can be attributed to the connection of the silane coupling agent KH-570, which contains a C=C double bond, to the surface of SiO_2_ particles. Subsequently, during radical polymerization, a protective film forms on the particle surface, resulting in a higher solid content of silica aerogel and, consequently, an increased density. For subsequent experiments, a volume ratio of 10:1 for silicic acid solution to KH-570 was chosen for performance analysis.

As illustrated in Figure 8, the density of silica aerogel exhibits an initial decrease followed by an increase as the dosage of MTMS is increased. At a volume ratio of 1:2 for the silicic acid solution to MTMS, the minimum density is recorded as 184 mg/cm^3^. Conversely, at a volume ratio of 1:5, the maximum density reaches 282 mg/cm^3^. This can be attributed to the hydrolysis of MTMS, which leads to the formation of -OH and -CH_3_ groups. During the polycondensation reaction, hydrophobic methyl groups infiltrate the gel matrix, enhancing the structure of silica aerogel and resulting in a decrease in density. However, when the MTMS content becomes excessive, some sol ions fail to effectively cross-link with -OH, causing an increase in the density of silica aerogel. For subsequent experiments, a volume ratio of 1:2 between silica solution and MTMS was selected for performance analysis.

### 3.3. Analysis of Surface Enhancement and Modification Mechanism

#### 3.3.1. KH-570 Modification Mechanism

The molecule of 3-methacryloxypropyl Trimethoxy silane (KH-570) contains the -OCH_3_ group which, after hydrolysis, can undergo a single displacement reaction with the -OH group on the gel surface. This reaction contributes to an increase in the structural strength of silica aerogel. Additionally, apart from the -OCH_3_ group, the C=C in the KH-570 molecule can undergo radical polymerization when a radical initiator is introduced. The introduction of species reintroduction polymers further enhances the performance of aerogel through KH-570 modification. During the modification process, KH-570 is typically mixed with anhydrous ethanol first and then combined with a silicic acid solution. This gradual mixing helps slow down the reaction rate and ensures a more uniform cross-linking of sol particles, thereby reducing the capillary force of silica aerogel [37,38]. 

KH-570 is utilized to reinforce and modify silica aerogel, and the polycondensation process involves two scenarios: with water and anhydrous conditions. In the presence of water, KH-570 undergoes hydrolysis initially, followed by a condensation reaction with the hydroxyl group (Si-OH) on the surface of the wet gel. Simultaneously, the hydrolyzed KH-570 also undergoes a condensation reaction itself. The chemical reaction equations involved in this process are denoted as 5~7 [39].


(5)

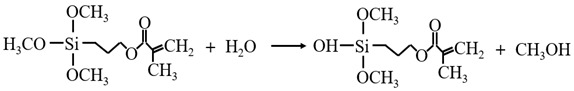




(6)

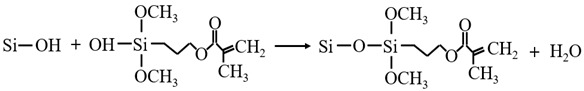




(7)

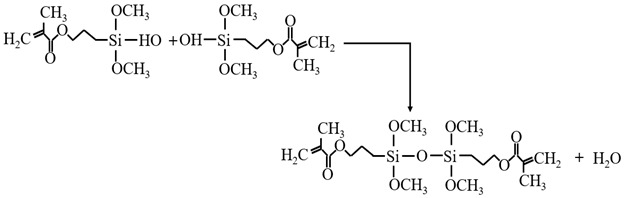



In the absence of water, KH-570 undergoes a condensation reaction directly with Si-OH. During the experiment, anhydrous ethanol is employed to facilitate the removal of water from the wet gel matrix. Consequently, KH-570 primarily reacts with the silicon hydroxyl groups on the surface of the wet gel through a condensation reaction in the absence of water.


(8)

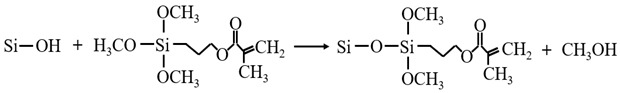



#### 3.3.2. MTMS Modification Mechanism

**Figure 9:**
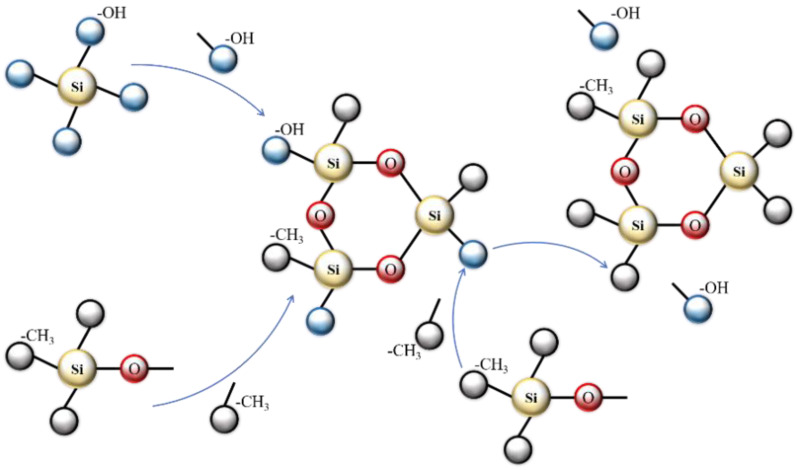
MTMS-enhanced modified silica aerogel mechanism diagram [40].

Three -OCH_3_ groups in the molecules of alkyl tri alkoxysilane (MTMS) undergo a reaction with -OH groups after hydrolysis. After the hydrolysis of water glass, each silicon atom carries four hydroxyl groups, while after MTMS hydrolysis, it carries an inert -CH_3_ group, which can condense under the influence of an alkaline catalyst. Due to the presence of the methyl group in MTMS after hydrolysis, the polycondensation reaction is slowed down, leading to the preferential formation of primary particles of a specific size through the polycondensation of Si(OH)_4_. As the reaction progresses, the hydrolysis product CH_3_-Si(OH)_3_ of MTMS also participates in the polycondensation reaction, resulting in a significant amount of unreacted Si-OH on the surface of the primary particles. Through the reaction with the primary particles, the -OH group is replaced, and the -CH_3_ group is successfully grafted onto the gel skeleton. As the condensation reaction continues, the primary particles grow into secondary particles, and the -OH groups on the surface are continuously replaced by -CH_3_ groups [40] (show in Figure 9).

### 3.4. Effects of Surface Enhancement Modification on the Structure and Properties of Silica Aerogel

#### 3.4.1. Phase Analysis

**Figure 10:**
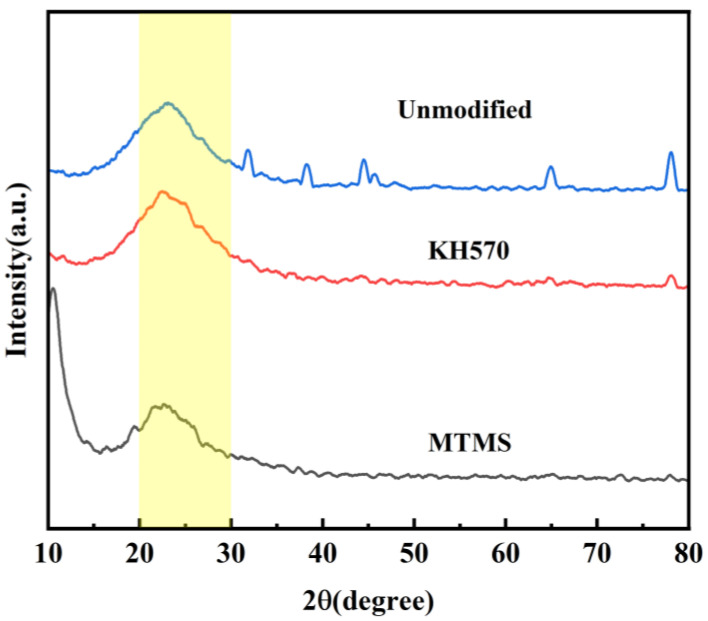
XRD patterns of polymer-reinforced modified silica aerogel.

In order to investigate the impact of various strengthening modifiers on the crystal structure of silica aerogel, X-ray diffraction (XRD) analysis was performed on the KH-570, MTMS-modified, and unmodified silica aerogel. The results of this analysis are depicted in Figure 10.

In Figure 10, it can be observed that the XRD patterns of the aerogel samples under the three different modification conditions exhibit prominent peak broadening in the range of 20~30°, indicating a predominantly amorphous structure of silicon in the samples. The unmodified samples display diffraction peaks corresponding to LiH and Fe_2_O_3_, which can be attributed to the presence of high levels of impurities in the water glass. Despite the use of a strong acidic cation exchange resin during pre-treatment, the complete removal of metal ions could not be achieved. On the other hand, no such peaks are observed after KH-570 and MTMS enhancement, suggesting that the modifiers might have undergone a coordination reaction with the metal ions in the water glass, forming compounds like metal silicates. These compounds typically have low solubility and tend to precipitate, resulting in a significant reduction in the metal ion content after modification.

#### 3.4.2. Physical Property Analysis

**Figure 11:**
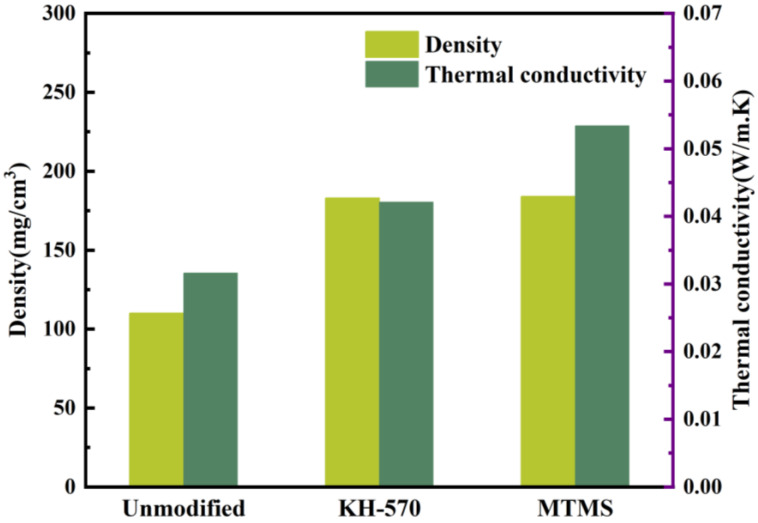
Effect of polymer-reinforced modification on density and thermal conductivity of SiO_2_.

In order to validate the modification mechanism of various strengthening modifiers, the thermal conductivity of KH-570- and MTMS-modified, as well as unmodified silica aerogel was measured, and its thermal insulation performance was analyzed. The results of this analysis are presented in Figure 11.

As depicted in Figure 11, both the density and thermal conductivity of the unmodified silica aerogel are low, measuring 110 mg·cm^−3^ and 0.0316 W·(m·K)^−1^, respectively. However, when enhanced with KH-570, the density and thermal conductivity of the silica aerogel increase significantly to 183 mg·cm^−3^ and 0.0421 W·(m·K)^−1^, respectively. While the density of the MTMS-enhanced silica aerogel is not considerably different from the KH-570-enhanced version, there is an improvement in the thermal conductivity. The density of the silica aerogel modified with MTMS is measured at 184 mg·cm^−3^, while its thermal conductivity is 0.0534 W·(m·K)^−1^.

**Figure 12:**
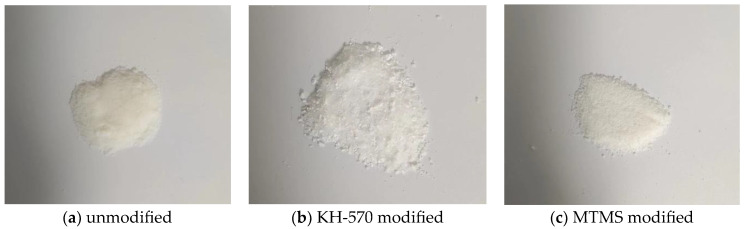
Diagram of different reinforced modified silica aerogel.

Figure 12 presents the visual representation of the reinforced and modified silica aerogel after 30 min of grinding. Based on the density and thermal conductivity of the silica aerogel, it can be observed that the order is as follows: MTMS > KH-570 > unmodified. However, in terms of appearance, all samples appear as powder, with the unmodified samples lacking any discernible particle sensation, having smaller particle sizes and exhibiting poorer mechanical properties during extrusion. Conversely, the samples modified with KH-570 and MTMS display a pronounced particle sensation, larger particle sizes, and higher hardness during extrusion. Although there is a slight increase in density and thermal conductivity for the reinforced and modified silica aerogel, it still falls within the low density and low thermal conductivity range. However, a significant improvement in mechanical properties is achieved. Therefore, strengthening and modification effectively enhance the mechanical properties of silica aerogel.

#### 3.4.3. Chemical Property Analysis

**Figure 13:**
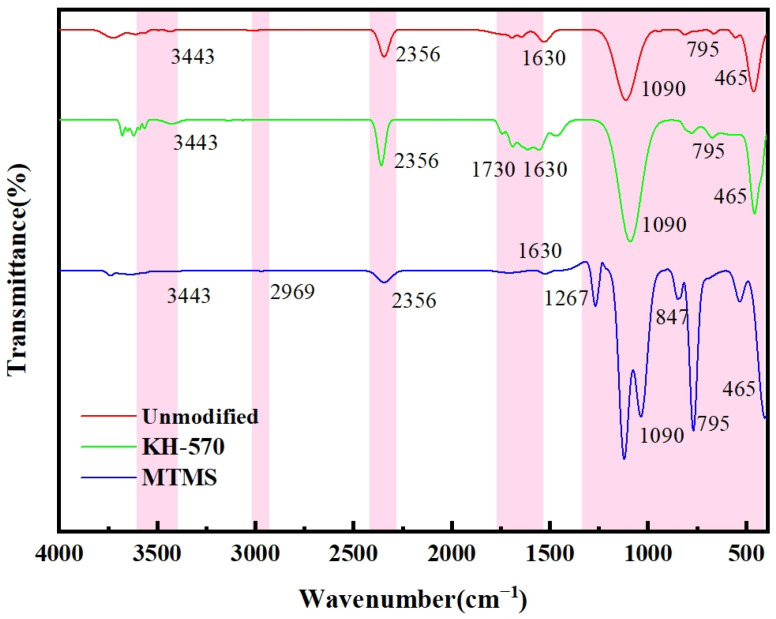
Infrared spectra of polymer-reinforced modified silica aerogel.

In order to validate the modification mechanism of various strengthening modifiers, the infrared spectra of KH-570- and MTMS-modified, as well as unmodified silica aerogel were measured. The functional groups present in the samples were then analyzed. The results of this analysis are depicted in Figure 13.

As illustrated in Figure 13, all samples exhibit distinct bands at approximately 465 cm^−1^, 795 cm^−1^, and 1090 cm^−1^. These bands correspond to the bending vibration, symmetric stretching vibration, and antisymmetric stretching vibration, respectively, of the Si-O-Si bond. These bonds form the skeleton structure of aerogel. Additionally, all samples display an absorption peak at 2356 cm^−1^, which is attributed to the stretching vibration of the C=O bond in CO_2_ present in the air. This peak may be influenced by the testing environment.

The spectra also reveal bands at 2969 cm^−1^, 1267 cm^−1^, and 847 cm^−1^, representing the antisymmetric stretching vibration peak, antisymmetric bending vibration peak, and symmetric bending vibration peak, respectively, of the -CH_3_ group.

The bands near 3443 cm^−1^ and 1630 cm^−1^ indicate the presence of -OH and H-O-H infrared bands. However, the peak intensity is weak due to the replacement of Si-OH on the surface of the modified aerogel with Si-CH_3_, thus enhancing the hydrophobicity of the aerogel.

In the spectrum of the aerogel modified with KH-570, bands are observed at 1630 cm^−1^ and 1730 cm^−1^. The bands at 1630 cm^−1^ may be attributed to the presence of adsorbed water or the bending vibration of the C=C bond. The absorption peak at 1730 cm^−1^ corresponds to the stretching vibration absorption peak of the C=O bond, indicating the successful bonding of KH-570 to the surface of silica aerogel [38,41].

#### 3.4.4. Analysis of Pore Structure and Specific Surface Area

**Figure 14:**
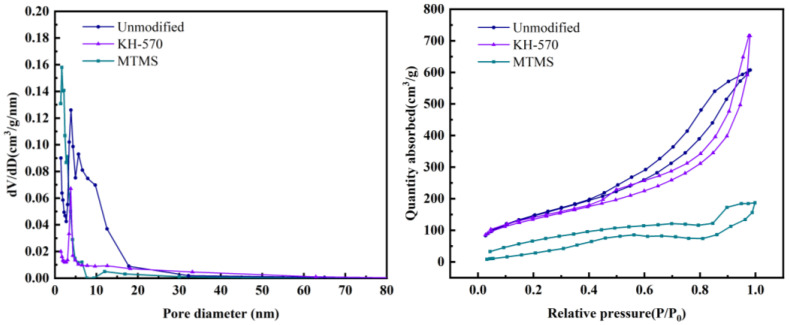
N_2_ adsorption–desorption isotherms and pore size distribution curves of different enhanced modified silica aerogels.

In order to investigate the impact of different reinforcement phases on the specific surface area and pore structure of silica aerogel, the unmodified and KH-570- and MTMS-modified silica aerogels were analyzed using a specific surface area analyzer (BET). The porosity results are presented in Table 6, while the N_2_ adsorption–desorption contour line, temperature, and related parameters, as well as the pore size distribution curve, are depicted in Figure 14.

As depicted in Figure 14, the adsorption–desorption isotherm curves of the unmodified, MTMS-modified, and KH-570-modified silica aerogel samples all exhibit Type IV behavior, indicating that the prepared silica aerogel is a mesoporous material. The unmodified and KH-570-modified silica aerogels show an H3-type hysteresis loop in the middle- and high-pressure range, suggesting the presence of a slit-like mesoporous structure. On the other hand, the MTMS-modified silica aerogel displays an H2 (b)-type hysteresis loop in the middle- and high-pressure range, indicating a relatively wider pore size distribution and potential channel blockage [37].

Under the three modification conditions, the N_2_ adsorption capacity of the aerogel is 715 cm^3^·g^−1^ after KH-570 modification, 607 cm^3^·g^−1^ for the unmodified sample, and 187 cm^3^·g^−1^ after MTMS modification. From the pore size distribution curve, the pore size ranges for the unmodified, KH-570-modified, and MTMS-modified aerogel samples are 2.7~20 nm, 2.5~15 nm, and 1.5~20 nm, respectively. The most probable diameters are 3.865 nm, 3.82 nm, and 1.661 nm.

Table 6 reveals that the maximum specific surface area of the unmodified silica aerogel is 538.7 m^2^·g^−1^. The specific surface area and pore volume of the KH-570-modified and MTMS-modified aerogel are lower than the unmodified sample, measuring 487.9 m^2^·g^−1^ and 146.8 m^2^·g^−1^, respectively. The maximum pore volume for the KH-570-modified aerogel is 1.107 cm^3^·g^−1^, while the minimum pore volume for the MTMS-modified silica aerogel is 0.425 cm^3^·g^−1^.

In summary, the performance of the KH-570-modified silica aerogel is superior to that of the MTMS-modified silica aerogel, and the modified aerogel exhibits a slightly lower performance compared to the unmodified aerogel. However, considering previous research, reinforcement modification enhances the skeleton structure and improves the mechanical properties. Therefore, selecting KH-570 as the reinforcement modifier can lead to a better specific surface area, pore volume, and stronger mechanical properties.

#### 3.4.5. Hydrophobicity Analysis

**Figure 15:**
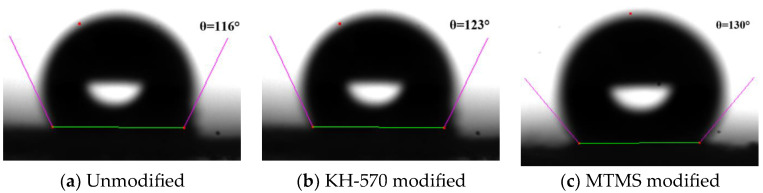
Contact angle of different reinforced modified silica aerogel.

In order to examine the impact of different reinforcement phases on the hydrophobicity of silica aerogel, contact angle analysis was conducted on the unmodified, KH-570-modified, and MTMS-modified samples using a contact angle measurement. The experimental results are presented in Figure 15.

As depicted in Figure 15, the contact angles measured for the three samples indicate hydrophobic silica aerogel properties, with values of 116°, 123°, and 130°, respectively. The presence of residual Si-OH hydrophilic groups in the unmodified silica aerogel may explain the slight hydrophilicity observed, suggesting that the complete conversion of Si-OH to Si-CH_3_ was not achieved with HMDSO. However, the enhancement and modification with KH-570 and MTMS further replaced the unmodified Si-OH groups with Si-CH_3_, leading to improved hydrophobic properties. The hydrophobicity of the three samples can be ranked in descending order as follows: MTMS > KH-570 > Unmodified.

## 4. Conclusions

1. The impact of different acid leaching conditions on the desilication rate of fly ash can be ranked as follows: hydrochloric acid concentration > solid–liquid ratio > reaction temperature > reaction time. The hydrochloric acid concentration and solid–liquid ratio were found to have the most significant effects on the desilication rate.
2. The optimal conditions for acid leaching were determined as follows: a hydrochloric acid concentration of 20 wt%, a solid–liquid ratio of 1:4, a reaction time of 2 h, and a reaction temperature of 100 °C.
3. After the calcination and activation of fly ash using sodium carbonate, the highest desilication rate of 81.72% was achieved with an acid-leaching solid-to-liquid ratio of 1:7.
4. Silica aerogel was prepared by ambient pressure drying using a sodium silicate solution obtained from fly ash as a silicon source. KH-570 and MTMS were used as strengthening modifiers. The lowest density of silica aerogel, 183 mg·cm^−3^, was obtained with a volume ratio of silica solution to KH-570 of 10:1. Likewise, the lowest density of silica aerogel, 184 mg·cm^−3^, was achieved with a volume ratio of silicic acid solution to MTMS of 1:2. And after characterizing by XRD, FTIR, and BET, the KH-570-enhanced aerogel exhibited the best overall performance.
5. The optimal performance parameters of silica aerogel were characterized by XRD, FTIR, and BET, resulting in thermal conductivity, a specific surface area, pore volume, average pore size, and contact angle values of 0.0421 W·(m·K)^−1^, 487.9 m^2^·g^−1^, 1.107 cm^3^·g^−1^, 9.075 nm, and 123° respectively.

## Figures and Tables

**Table 1:** Chemical composition of fly ash.

Component (wt%)	SiO_2_	Al_2_O_3_	Fe_2_O_3_	CaO	MgO	Na_2_O	K_2_O	SO_3_	MnO	TiO_2_	Loss
Fly ash	50.04	33.52	6.60	4.16	0.33	0.02	1.33	0.94	0.075	1.48	1.52

**Table 2:** Orthogonal experimental design for the influence of different reaction conditions on the desilication rate of fly ash.

Level Number of Group	HCl/(wt%)	Solid–Liquid Ratio	Reaction Time/h	Reaction Temperature/°C
1	15%	1:3	2 h	90 °C
2	20%	1:4	3 h	100 °C
3	25%	1:5	4 h	110 °C

**Table 3:** Analysis table of orthogonal test.

Test Group Number	Factor				Desilication Rate/%
	A	B	C	D	
1	15%	1:3	2 h	90 °C	34.99
2	15%	1:4	3 h	100 °C	41.19
3	15%	1:5	4 h	110 °C	41.5
4	20%	1:3	3 h	110 °C	35.7
5	20%	1:4	4 h	90 °C	39.88
6	20%	1:5	2 h	100 °C	42.67
7	25%	1:3	4 h	100 °C	32.58
8	25%	1:4	2 h	110 °C	37.28
9	25%	1:5	3 h	90 °C	33.19

Note: A represents the factor of HCl concentration, B represents the factor of Solid–liquid ratio, C represents the factor of reaction time, and D represents the factor of reaction temperature.

**Table 4:** Orthogonal test results analysis table.

Level	A	B	C	D
1	15%	1:3	2 h	90 °C
2	20%	1:4	3 h	100 °C
3	25%	1:5	4 h	110 °C
Extreme deviation	5.07	5.03	1.62	2.79

Note: A represents the factor of HCl concentration, B represents the factor of Solid–liquid ratio, C represents the factor of reaction time, and D represents the factor of reaction temperature.

**Table 5:** Surface-enhanced modified silica aerogel solution proportioning.

Serial Number	Silicon Source	Modified Liquid 1		Modified Liquid 2	
	Silicic Acid Solution/mL	KH-570/mL	EtOH/mL	MTMS/mL	EtOH/mL
1	10	5	20	10	40
2	10	4	16	20	80
3	10	3	12	30	120
4	10	2	8	40	160
5	10	1	4	50	200

**Table 6:** Effect of different enhancement modifications on the pore properties of silica aerogel.

Sample	Specific Surface Area/(m^2^/g)	Pore Volume/(cm^3^/g)	Average Pore Diameter/(nm)
Unmodified	538.7	0.991	7.358
KH-570	487.9	1.107	9.075
MTMS	146.8	0.425	11.58

## Data Availability

Data are contained within the article and Supplementary Materials.

## Supplementary Materials

The following supporting information can be downloaded at: https://www.mdpi.com/article/10.3390/ma17071614/s1, Table S1: Experimental reagents. Table S2: Main experimental equipment. Table S3: Orthogonal test results analysis table.
