# Supplementary material for: Synthesis and Surface Strengthening Modification of Silica Aerogel from Fly Ash
Source: Materials (Basel). 2024 Apr 1;17(7):1614. doi: 10.3390/ma17071614 (PMC11012301; doi:10.3390/ma17071614)
Supplement: Supplementary file 1 [file materials-17-01614-s001.zip › materials-2869229-supplementary.pdf]

Supplementary Materials:

1.1 Reagents

The main reagents used in the experiment are shown in Table S1.

Table S1. Experimental reagents.

Reagents	Specifications	Suppliers
Na_2CO_3	AR	Aladdin Reagent Shanghai Co., Ltd
HCl	AR	China National Pharmaceutical Group Chemical Reagent Co., Ltd
C_9H_{12}	AR	Aladdin Reagent Shanghai Co., Ltd
NaOH	AR	Aladdin Reagent Shanghai Co., Ltd
$NH_3{\cdot}H_2O$	AR	Aladdin Reagent Shanghai Co., Ltd
CH_3CH_2OH	AR	Aladdin Reagent Shanghai Co., Ltd
$CH_3Si\ (CH_3O)_3$	98%	Aladdin Reagent Shanghai Co., Ltd
$CH_3(CH_2)_3CH_3$	AR	Aladdin Reagent Shanghai Co., Ltd
$((CH_3)_3Si)_2O$	98%	Aladdin Reagent Shanghai Co., Ltd
$C_6H_{16}O_3Si$	>98%	Aladdin Reagent Shanghai Co., Ltd

1.2 Equipment

The model numbers of the equipment used for testing are shown in Table S2.

Table S2. Main experimental equipment.

Equipment	Model	Manufacturer
X-ray diffraction spectrometer	XD-3X	Beijing Puxi General Instrument Co., Ltd
Physical adsorption analyzer	ASAP2020	Mike Company in the United States
FT-IR	VERTEX70	Bruker, Germany
Thermal conductivity meter	TC-3000E	Xi'an Xiaxi Electronic Technology Co., Ltd
Contact angle tester	CA100B	Shanghai Yingnuo Precision Instrument Co., Ltd

Table S3. Orthogonal test results analysis table.

Item	Level	A	B	C	D
K	1	117.68	103.27	114.94	108.06
	2	118.25	118.35	110.08	116.44
	3	103.05	117.36	113.96	114.48
k	1	39.23	34.42	38.31	36.02
	2	39.42	39.45	36.69	38.81
	3	34.35	39.12	37.99	38.16
Optimal Level		2	2	1	2
Range		5.07	5.03	1.62	2.79
Number of levels		3	3	3	3
Number of repetitions per level		3	3	3	3

Note: K represents the sum of the desilication rates under the same level and condition in the experiment. Meanwhile, k represents the average of the summed desilication rates. Range indicates the range of the factors in the experiment, calculated as follows for the same factor (k_1-k_3): $R = k_{max} - k_{min}$.
